# From Circuits to Symphonies: A Systems‐Engineering Blueprint for Multimicrobial Synthetic Biology

**DOI:** 10.1002/advs.76981

**Published:** 2026-07-31

**Authors:** Miguel Fernández‐Niño, Ludger A. Wessjohann, José Manuel Guillamón, Daniela Burgos‐Toro, Silvia Moriano‐Gutiérrez, Giacomo Di Matteo, Zia‐ul Islam, Rinke van van Tatenhove‐Pel, Vanessa Rossetto Marcelino, Rodrigo Ledesma‐Amaro

**Affiliations:** ^1^ Department of Biotechnology Institute of Agrochemistry and Food Technology (IATA‐CSIC) Valencia Spain; ^2^ Department of Bioorganic Chemistry Leibniz‐Institute of Plant Biochemistry (IPB) Halle (Saale) Germany; ^3^ Facultad de Ciencias Agropecuarias. Universidad de Cundinamarca Fusagasugá Colombia; ^4^ Department of Chemistry and Technology of Drugs Sapienza Università di Roma Rome Italy; ^5^ School of Science and the Environment Memorial University Corner Brook NL Canada; ^6^ Department of Biotechnology Delft University of Technology Delft The Netherlands; ^7^ Department of Microbiology and Immunology at the Peter Doherty Institute for Infection and Immunity University of Melbourne VIC Australia; ^8^ Department of Bioengineering and Imperial College Centre for Synthetic Biology Imperial College London London UK; ^9^ Bezos Centre for Sustainable Protein Imperial College London London UK; ^10^ UKRI Mission Hub on Microbial Food Imperial College London London UK

**Keywords:** microbial consortia, multicellular systems engineering, synthetic biology, synthetic ecology, systems biotechnology

## Abstract

Synthetic biology is expanding from single‐species engineering toward the design of synthetic microbial consortia capable of distributed metabolism, coordinated behavior, and enhanced functional robustness. However, engineering frameworks derived from intracellular genetic circuits remain insufficient to capture the ecological, temporal, and spatial dynamics that govern multispecies systems. Here, a symphony‐inspired systems‐engineering framework is presented for multimicrobial synthetic biology, organizing consortium design across four application‐dependent coordination layers: communication, temporal regulation, ecological structuring, and predictive modeling. Building on this framework, a modular engineering blueprint is proposed to guide the rational selection and integration of coordination layers according to engineering objectives, translating systems‐level principles into operational strategies for the construction, stabilization, and optimization of synthetic microbial consortia. The manuscript integrates advances in interspecies signaling, oscillatory control, synthetic ecology, hybrid modeling, and real‐time monitoring while addressing constraints including metabolite transport, population drift, parameter uncertainty, and process‐scale implementation. The proposed framework is modular rather than prescriptive, allowing different coordination layers to be deployed according to application requirements and environmental complexity. By repositioning multimicrobial engineering as a problem of coordinated systems design rather than isolated genetic control, this Perspective provides a structured foundation for the development of synthetic microbial communities across industrial, environmental, and therapeutic applications.

## Graphical abstract

Engineering synthetic microbial consortia requires moving beyond intracellular genetic circuits toward coordinated multicellular design. A modular systems‐engineering framework is presented that integrates communication, temporal coordination, ecological structuring, and predictive modeling to guide application‐dependent consortium design, implementation, and optimization across diverse biotechnological applications.

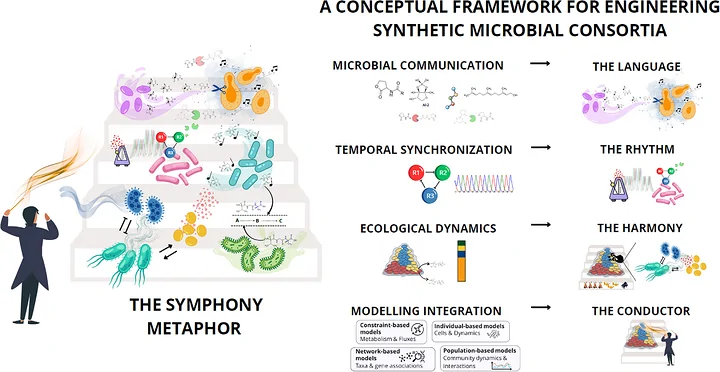

## 1 Introduction: From Unicellular to Multicellular Synthetic Biology

Synthetic biology has evolved from its early focus on the engineering of single microbial species into a broader discipline aimed at designing and controlling increasingly complex biological systems [1, 2, 3, 4, 5]. The formative years of the field were dominated by tractable chassis such as *Escherichia coli*, *Corynebacterium glutamicum*, *Bacillus subtilis*, *Saccharomyces cerevisiae*, and *Pichia pastoris*, which enabled the development of foundational genetic circuits, biosensors, and metabolic pathways [4, 6, 7]. These monoculture‐based systems were instrumental in establishing the design‐build‐test‐learn framework and the engineering principles that underpin modern synthetic biology [6]. However, they remain insufficient to capture the emergent behaviours, distributed metabolism, and ecological interactions that characterize microbial life in natural and industrial environments [8, 9].

In nature, microorganisms rarely exist in isolation. Instead, they form self‐organized communities shaped by competitive and cooperative interactions including syntrophy, cross‐feeding, mutualism, and spatial structuring [8, 10]. Reproducing such properties in engineered systems requires moving beyond single‐strain optimization toward the rational design of synthetic microbial consortia [3, 5, 11]. Unlike monocultures, multispecies systems can distribute metabolic tasks across specialized partners, enabling functionalities that are difficult or impossible to encode within a single organism. This functional partitioning has stimulated growing interest across biomanufacturing, environmental biotechnology, and living therapeutics [1, 12, 13, 14]. Division of labour can reduce metabolic burden and improve pathway modularity, cooperative degradation pathways can expand substrate utilization, and engineered microbial communities may achieve greater robustness and adaptability than single‐strain systems.

Despite these opportunities, designing synthetic consortia with predictable functionality remains a major engineering challenge. Unlike intracellular genetic circuits, multicellular systems must coordinate multiple interacting layers simultaneously, including communication, temporal dynamics, ecological structuring, and environmental context [3, 5, 11]. These interactions are strongly shaped by metabolite exchange, spatial organization, diffusion constraints, stochastic fluctuations, and population‐level feedback loops [15]. Consequently, engineering principles derived from single‐cell circuit logic are often insufficient to predict or stabilize consortium‐level behaviour.

**FIGURE 1:**
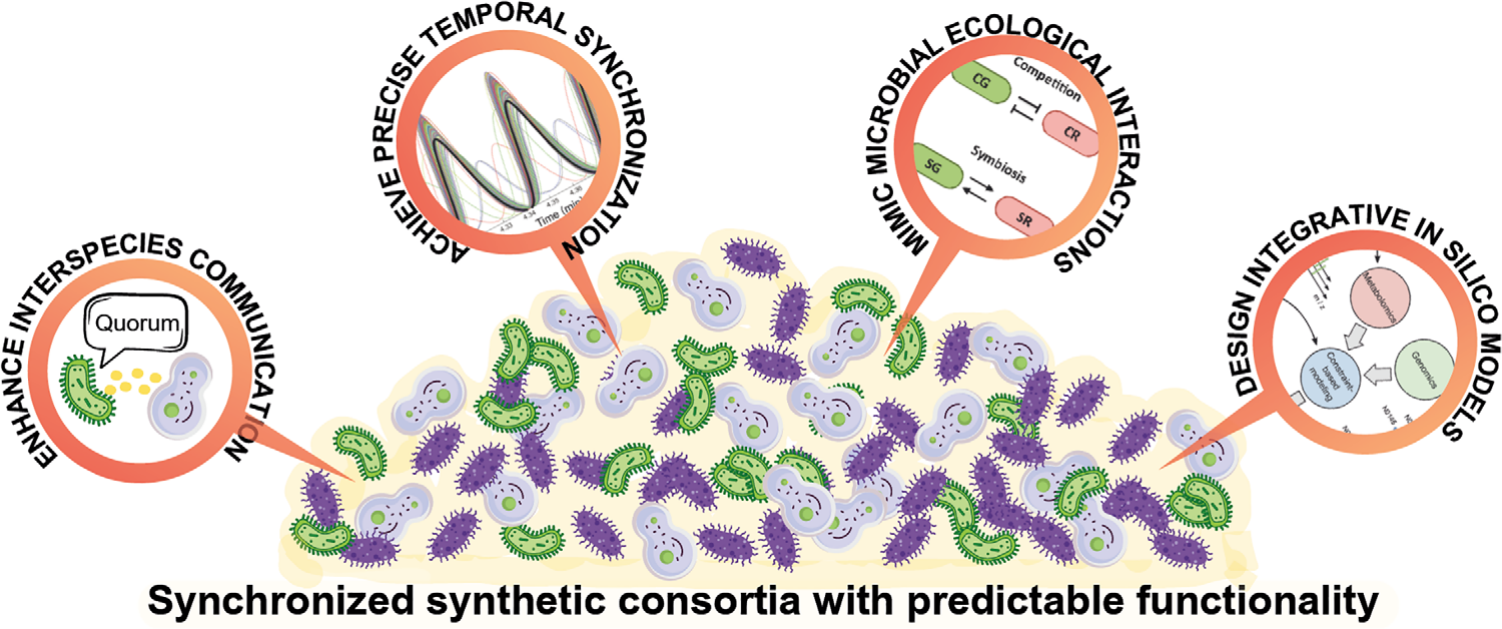
Systems‐level coordination layers in synthetic microbial consortia engineering. Schematic representation of four major coordination dimensions that can contribute to consortium design: (i) interspecies communication mediated by quorum sensing and related signalling networks; (ii) temporal coordination enabled by oscillatory circuits or environmentally driven dynamics; (iii) ecological structuring through niche partitioning, metabolite exchange, spatial organization, and controlled population interactions; and (iv) integrative computational modelling linking metabolic fluxes, interspecies exchange, and ecological dynamics. Rather than representing universally required components, these layers define a modular engineering design space whose implementation depends on the functional objectives, environmental complexity, and desired level of system control. The interconnected background highlights the continuous exchange of metabolites, signals, and ecological feedbacks that collectively shape community‐level behaviour.

Figure 1 summarizes four major coordination layers that frequently govern synthetic consortia design: (i) programmable microbial communication, (ii) temporal coordination and synchronization, (iii) ecological structuring and coexistence, and (iv) integrative modeling frameworks capable of linking metabolic and ecological dynamics. Importantly, these layers should not be interpreted as universally mandatory components. Rather, they define a modular design space whose implementation depends on application complexity, environmental variability, and the degree of coordination required for a given function.

All these considerations highlight the need for engineering frameworks capable not only of integrating multiple coordination layers, but also of determining when, why, and to what extent each layer should be implemented according to specific engineering objectives. In this Perspective, advances in communication, temporal regulation, synthetic ecology, and integrative modeling are synthesized into a systems‐engineering framework for multimicrobial design. Building on this foundation, a modular engineering blueprint is proposed to support the rational selection, integration, and evaluation of coordination layers according to application‐specific requirements, environmental complexity, and operational constraints. The resulting framework provides a decision‐oriented approach for designing synthetic microbial consortia across industrial, environmental, and therapeutic applications.

## 2 From Circuits to Symphonies: A Systems‐Level Framing for Multimicrobial Engineering

Since its emergence, synthetic biology has been strongly influenced by the circuit paradigm, in which genes, promoters, and regulatory elements are conceptualized as modular biological components analogous to electronic devices [16, 17]. This framework enabled the standardization of biological parts and supported the development of predictable genetic circuits, biosensors, and engineered metabolic pathways in tractable single‐species chassis [16, 17, 18]. Circuit‐based logic proved highly effective for intracellular regulation and remains foundational for modern synthetic biology.

However, as the field increasingly expands toward multispecies systems, the limitations of purely circuit‐centered engineering become more apparent. Unlike intracellular constructs, microbial consortia operate through distributed interactions across populations, where system‐level behaviour emerges from interspecies communication, ecological feedback, metabolite exchange, spatial organization, and temporal dynamics [10, 19]. These properties are often adaptive, context‐dependent, and non‐linear, making them difficult to predict or stabilize using frameworks originally developed for isolated genetic modules. Consequently, engineering microbial communities requires moving beyond deterministic single‐cell control toward coordination strategies capable of selecting and integrating communication, ecological, temporal, and predictive dimensions according to application‐specific requirements.

To organize these additional layers of complexity, this Perspective adopts a symphony‐inspired systems‐engineering framework for multimicrobial design. Within this framework, microbial communities are viewed as distributed systems in which communication, temporal regulation, ecological structuring, and predictive modeling collectively shape community‐level functionality. The emphasis, therefore, shifts from isolated intracellular modules toward the coordination of interacting populations operating under dynamic ecological constraints.

**FIGURE 2:**
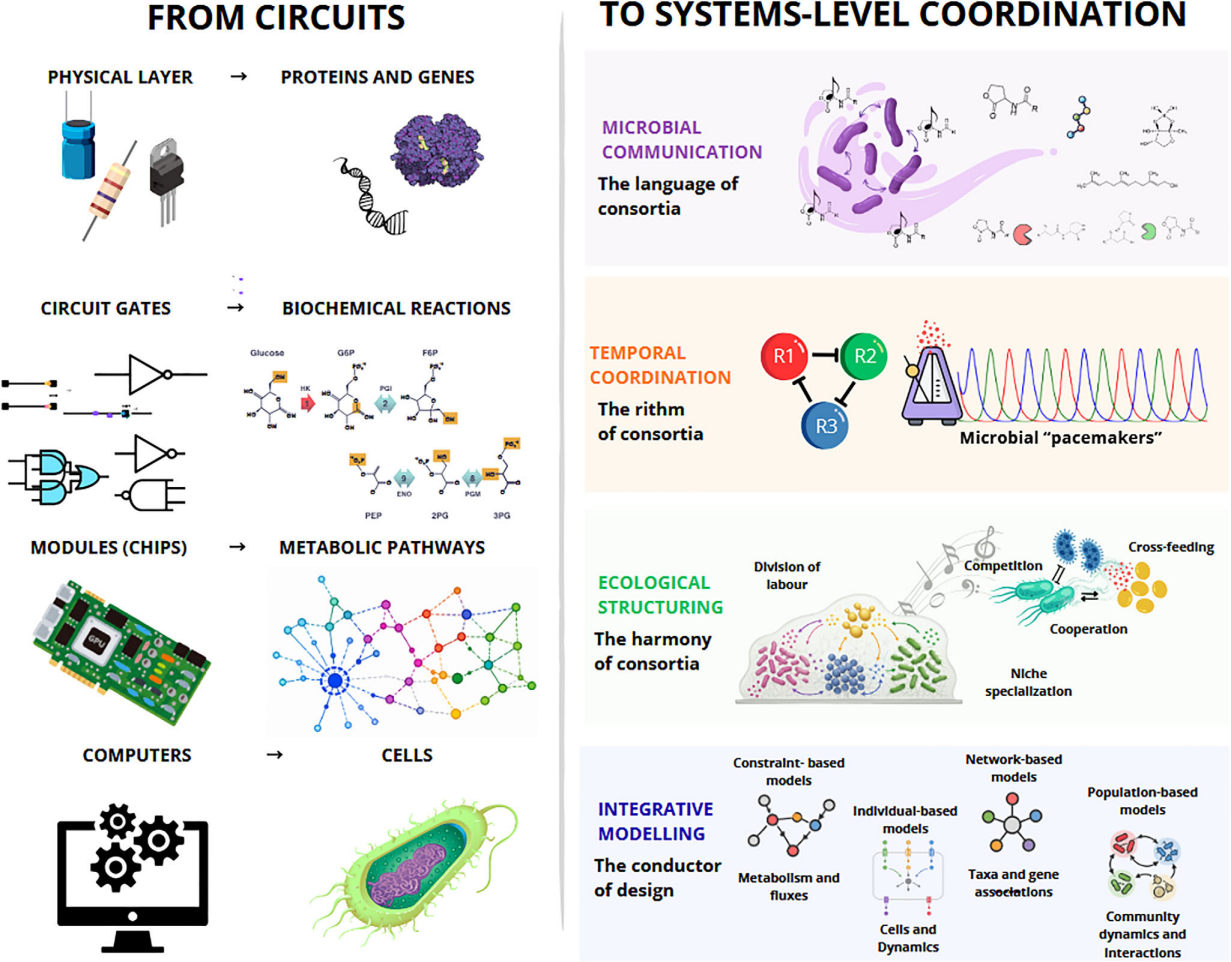
From intracellular circuits to systems‐level coordination in synthetic microbial consortia. The figure illustrates the transition from classical circuit‐based engineering, centered on intracellular control within a single chassis, toward the engineering of distributed multicellular systems operating across multiple organizational layers. Four major coordination dimensions frequently involved in consortium design are highlighted: microbial communication, temporal coordination, ecological structuring, and integrative modelling. Together, these coordination layers define a modular design space that supports the systems‐engineering framework developed throughout this Perspective. Importantly, they should not be interpreted as universally required components of all consortia, but rather as application‐dependent design dimensions whose selection and integration depend on specific engineering objectives, environmental complexity, and desired levels of control.

Figure 2 summarises this transition from circuit‐based engineering centered on intracellular determinism and chassis‐level control toward systems‐level coordination in synthetic microbial consortia. Building on this framing, the following sections examine four major coordination layers frequently involved in consortium engineering: communication (Section 3), temporal coordination (Section 4), ecological structuring (Section 5), and integrative modeling (Section 6). These concepts subsequently converge into the modular systems‐engineering framework presented in Section 7, where they are organized into a decision‐oriented blueprint for the selection, integration, and optimization of coordination layers in synthetic microbial consortia. Importantly, these coordination layers should be viewed as context‐dependent design dimensions whose relevance varies according to the functional objectives and complexity of the target system.

## 3 Communication as a Coordination Layer in Synthetic Consortia

Communication represents one of the principal coordination layers frequently used in synthetic microbial consortia, enabling distributed regulation across populations through the exchange of molecular, metabolic, or environmental information. Unlike intracellular genetic circuits, where regulatory interactions occur within a single cellular context, communication architectures in multispecies systems must coordinate responses across organisms with distinct growth rates, metabolic states, spatial distributions, and environmental sensitivities [20, 21, 22]. Importantly, communication should not be interpreted as a universally mandatory feature of all synthetic consortia. Some applications can operate effectively through ecological partitioning or passive metabolic coupling alone [23], whereas others require tightly coordinated signalling architectures to synchronize population‐level behaviours, regulate division of labour, or stabilize dynamic transitions between functional states. For example, engineered quorum‐sensing circuits have been used to regulate population composition, coordinate gene expression across strains, and control division of labour within synthetic consortia, demonstrating how communication can be deliberately engineered to control community‐level behaviour rather than emerge solely from ecological interactions [24, 25, 26].

Quorum sensing (QS) systems remain among the most widely used communication architectures in synthetic biology because they enable density‐dependent coordination through diffusible autoinducers coupled to regulatory responses [20, 21, 22]. LuxI/LuxR‐type systems and related signalling modules have been extensively adapted to control collective behaviours, synchronize pathway activation, and coordinate metabolic tasks across populations [24, 27, 28, 29]. Recent developments include multi‐channel architectures with tunable activation thresholds, orthogonal signalling modules, and synthetic autoinducer analogues with modified sensitivity and degradation kinetics [24, 27, 30, 31]. Quorum‐quenching mechanisms mediated by acylases, lactonases, or oxidoreductases further expand regulatory control by attenuating or degrading signalling molecules, thereby attenuating quorum‐sensing responses, modulating feedback intensity, and limiting signal propagation [32, 33]. These systems are particularly useful when synchronized population‐level activation, phased metabolic transitions, or coordinated switching between functional states are required. Notably, communication‐driven synchronization has been shown to support coherent oscillatory behaviour across spatially extended microbial populations, illustrating how communication architectures can also serve as a foundation for population‐level temporal coordination [34].

However, communication in synthetic consortia extends beyond canonical QS architectures. Metabolite‐mediated interactions through cross‐feeding represent an alternative strategy in which information exchange is embedded directly within metabolic fluxes. The exchange of amino acids, vitamins, cofactors, or central metabolic intermediates can generate mutual dependencies that stabilize community composition while simultaneously coupling coordination to resource allocation [35, 36]. Engineered amino acid auxotrophies, for example, have been used to maintain stable population structures and reduce competitive exclusion in complex environments such as the mammalian gut [35]. Likewise, synthetic consortia based on amino‐acid cross‐feeding have demonstrated that stable cooperation and population evenness can emerge without dedicated signalling circuits [35, 37]. Compared with dedicated signalling circuits, metabolite‐mediated communication may reduce engineering complexity by directly linking coordination to pathway activity, although these interactions are often more context‐dependent and difficult to decouple from environmental fluctuations.

Different engineering contexts may require communication strategies beyond canonical quorum sensing architectures. Contact‐dependent signalling systems, including type VI secretion systems and nanotube‐mediated exchange mechanisms, can support highly localized interactions in structured biofilms or spatially constrained environments [38, 39, 40]. Environmental sensing architectures allow populations to coordinate behaviour indirectly through shared responses to pH, oxygen gradients, nutrient depletion, redox shifts, or stress signals, thereby reducing the need for engineered signalling modules in fluctuating open systems [41]. Emerging CRISPR‐mediated intercellular communication platforms further extend the communication repertoire by enabling programmable transfer of genetic information or signal‐responsive transcriptional regulation across populations [42]. All these approaches considerably expand the range of coordination strategies available for multimicrobial engineering beyond traditional quorum sensing paradigms.

Despite these advances, engineering predictable communication across populations remains challenging. Signal diffusion, degradation kinetics, environmental variability, and stochastic fluctuations can alter activation thresholds and destabilize coordinated responses [43, 44]. Crosstalk between homologous regulators, incomplete orthogonality between signalling channels, and host‐specific metabolic interference may further compromise signal fidelity in multispecies environments [28, 29, 45, 46]. Spatial heterogeneity introduces additional constraints, as diffusion delays and uneven population distributions can desynchronize activation dynamics across biofilms or large‐scale bioprocesses. Moreover, communication architectures impose metabolic costs associated with signal synthesis, sensing, and regulatory burden, which may reduce stability under long‐term cultivation or industrial conditions. Addressing these limitations often requires insulated regulatory elements, engineered receptor specificity, dynamic feedback control, or spatial compartmentalization strategies capable of improving signal robustness and reducing unintended interactions [28, 29, 45, 46].

The selection of communication architectures, therefore, depends strongly on the functional objectives and environmental context of the consortium. QS‐based systems are particularly suitable for synchronized activation and coordinated population‐level transitions, whereas metabolite‐mediated interactions may be preferable when communication can be directly coupled to metabolic exchange. Contact‐dependent signalling becomes especially relevant in structured biofilms or surface‐associated communities, while environmental sensing strategies may reduce engineering burden in open or highly variable environments. Importantly, many successful consortia rely on combinations of these coordination modes rather than on a single communication strategy.

Beyond their signalling function, communication architectures regulate information flow, metabolic coupling, spatial organization, and collective behavioural transitions across interacting populations. Future advances will depend on expanding orthogonal communication repertoires, improving predictability across environmental scales, and integrating communication design with ecological structuring, temporal coordination, and modeling frameworks. These interactions become particularly important when communication must support synchronized or rhythmic behaviours across populations, as discussed in the following section on temporal coordination.

## 4 Temporal Coordination in Synthetic Consortia

Temporal coordination enables microbial communities to organize functions in time, synchronizing metabolic activities, behavioural transitions, and collective responses across interacting populations [47].Temporal regulation is commonly implemented through oscillatory or environmentally gated architectures capable of periodically modulating gene expression, metabolic activity, or intercellular signalling [44, 47, 48, 49]. These systems can coordinate sequential metabolic processes, synchronize stress responses, and temporally distribute biosynthetic tasks across interacting populations [50, 51, 52, 53]. Importantly, temporal coordination should not be interpreted as a universally required feature of all synthetic consortia. While some applications benefit substantially from synchronized or rhythmic regulation, others operate effectively through steady‐state ecological interactions or constitutive metabolic coupling alone.

The practical value of temporal control has been demonstrated in engineered consortia designed to generate synchronized population‐level oscillations through intercellular signalling networks, illustrating how community‐level synchronization can be deliberately engineered when required [34, 45]. Conversely, several successful synthetic consortia based on constitutive metabolic cross‐feeding and ecological stabilization have achieved robust functionality without requiring dedicated temporal control mechanisms [37, 54, 55].

Temporal coordination becomes particularly valuable when consortium functionality depends on sequential activation or phase‐dependent behaviour. In multispecies bioprocesses, temporal separation of incompatible metabolic phases can reduce intermediate accumulation, minimize metabolic interference, and improve pathway efficiency [34, 56]. Oscillatory activation may also distribute energetic burden across populations by alternating biosynthetic tasks over time rather than maintaining continuous pathway expression [57]. Proof‐of‐concept studies have further shown that such oscillatory architectures can coordinate gene expression across populations and sustain coherent temporal dynamics during multicellular operation [45, 58]. In other cases, rhythmic coordination enables synchronized metabolite release, staged substrate utilization, or dynamic adaptation to fluctuating environmental conditions such as nutrient pulses, oxygen oscillations, or stress exposure [59, 60, 61]. Environmentally responsive timing architectures, including light‐ or temperature‐gated systems, further expand the ability of synthetic consortia to dynamically adjust behaviour under heterogeneous conditions [62, 63].

Effective temporal coordination in microbial consortia frequently depends on coupling oscillatory behaviour with communication networks capable of synchronizing responses across populations. Quorum sensing systems, diffusible metabolites, or environmentally shared signals often function as synchronization layers that align oscillatory dynamics between cells or species [49, 50, 64, 65]. However, synchronization across spatially distributed populations remains highly sensitive to diffusion constraints, response kinetics, and environmental heterogeneity. Notably, Kim et al. demonstrated that long‐range temporal coordination can be achieved in spatially extended microbial consortia through positive‐feedback‐mediated signal propagation, highlighting both the potential and the engineering challenges associated with maintaining synchronized behaviour beyond local diffusion distances [34].

Signal propagation delays can introduce phase lags between subpopulations, while differences in growth rates or metabolic activity may progressively destabilize rhythmic coherence across the consortium. Asynchrony can lead to amplitude damping, desynchronization, or oscillation collapse, particularly under large‐scale or structured cultivation conditions [66, 67]. In coordinated metabolic systems, temporal desynchronization can result in asynchronous metabolite release, inefficient substrate channeling, unstable transitions between functional states, or decoupling of division‐of‐labour architectures [68, 69, 70]. Oscillation instability may additionally increase energetic waste through premature pathway activation or prolonged maintenance of non‐productive metabolic states [68, 71]. These effects become particularly relevant in industrial‐scale bioprocesses, where mixing limitations, spatial heterogeneity, and delayed signal propagation can amplify synchronization decay across large microbial populations.

Engineering robust temporal coordination therefore requires addressing multiple practical constraints simultaneously. Stochastic fluctuations in gene expression, diffusion limitations, environmental interference, and evolutionary drift can progressively destabilize oscillatory behaviour during long‐term cultivation [49, 50, 64, 65]. Increasing synchronization complexity may also impose substantial metabolic burden due to the energetic costs associated with signal production, sensing, and dynamic regulatory control. In some cases, excessive coordination layers may reduce rather than improve system stability, particularly when oscillatory architectures become overly sensitive to environmental perturbations or parameter variability. Consequently, more temporal regulation does not necessarily translate into better consortium performance.

**FIGURE 3:**
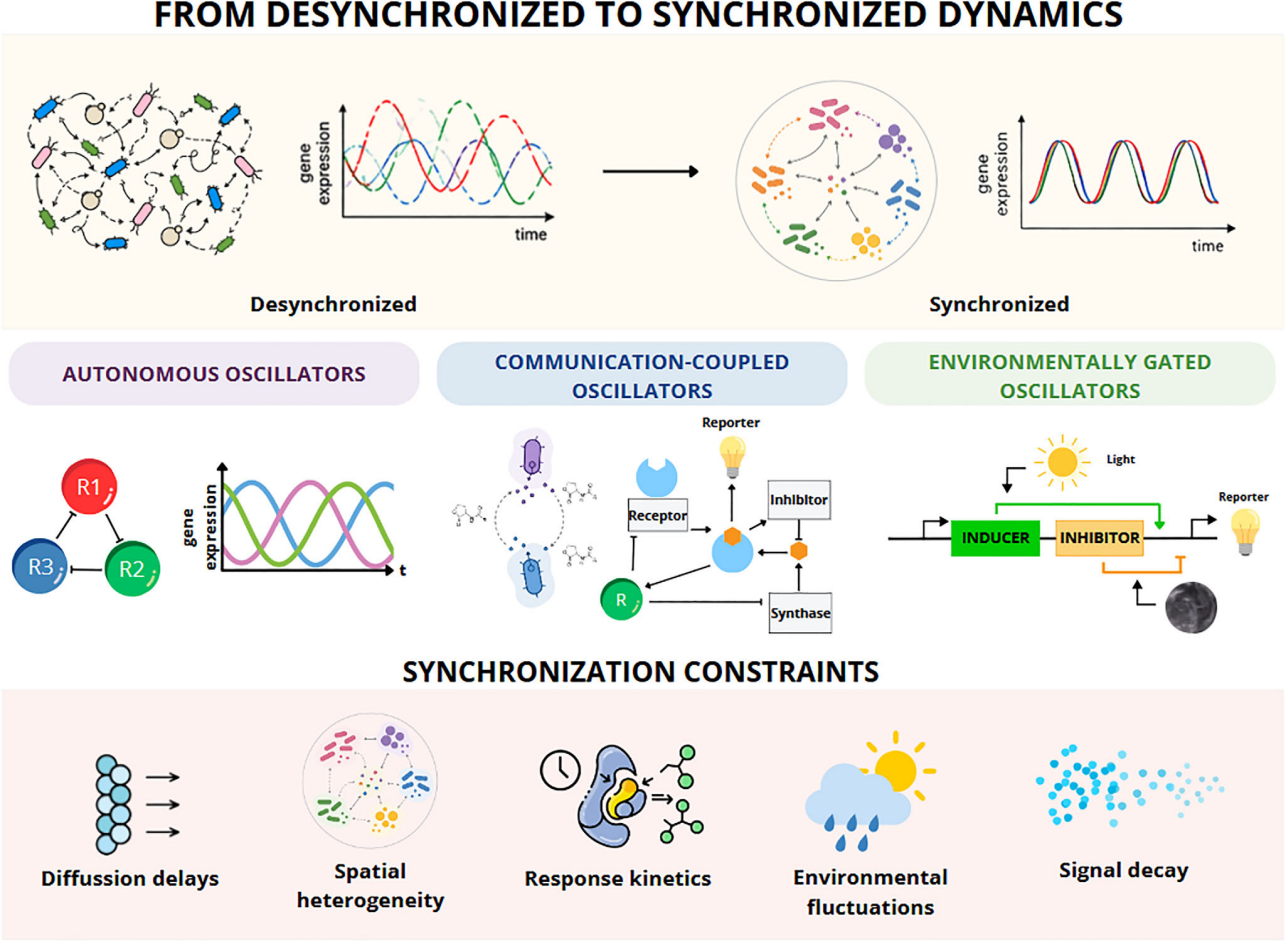
Temporal coordination architectures and synchronization constraints in synthetic microbial consortia. Conceptual representation of three major temporal coordination strategies frequently used in consortium engineering: autonomous oscillators, communication‐coupled oscillators, and environmentally gated oscillators. These architectures can support synchronized population behaviour, periodic gene expression, sequential pathway activation, and adaptive responses to environmental cues. The figure also illustrates the transition from desynchronized to synchronized community dynamics and highlights key engineering constraints that influence temporal robustness, including diffusion delays, spatial heterogeneity, response kinetics, environmental fluctuations, and signal decay. Together, these factors determine the stability, coherence, and scalability of temporally coordinated behaviours across microbial populations.

Several engineering strategies have been proposed to improve temporal robustness in synthetic consortia. Negative feedback architectures, delayed inhibition loops, dynamic gating systems, and environmental entrainment mechanisms can help stabilize oscillatory coherence and reduce stochastic drift [49, 50, 64, 65]. Spatial compartmentalization and microenvironmental structuring may additionally improve synchronization by limiting diffusion heterogeneity across populations [72]. Hybrid approaches coupling oscillatory control with ecological stabilization strategies are also increasingly explored to maintain rhythmic behaviour while preserving long‐term community stability [73, 74]. Figure 3 summarizes representative temporal coordination architectures together with the principal synchronization constraints and environmental factors influencing rhythmic coherence across microbial populations.

Nevertheless, many synthetic consortia may not require explicit temporal coordination. Systems based primarily on constitutive cross‐feeding, ecological niche partitioning, or spatial gradients can often achieve stable functionality without synchronized oscillatory control. Representative examples include amino‐acid cross‐feeding consortia, fungal‐bacterial bioconversion systems, and synthetic gut communities stabilized through metabolic interdependencies, all of which achieved stable coexistence and productive functionality without explicit temporal synchronization mechanisms [35, 54, 55]. In continuous bioprocesses or highly stable environments, steady‐state regulation may outperform dynamic architectures by reducing metabolic burden and minimizing synchronization failure risks [75]. Similarly, some applications may benefit more from ecological robustness than from tightly regulated temporal behaviour. These considerations highlight that temporal coordination should be selected according to operational necessity rather than incorporated as a default design objective.

## 5 Synthetic Ecology: Ecological Structuring and Stability in Engineered Consortia

Ecological structuring represents another major coordination layer frequently used in synthetic microbial consortia to stabilize population dynamics, maintain functional coexistence, and regulate distributed metabolic interactions. However, as with communication and temporal coordination, the degree of ecological engineering required strongly depends on application context, environmental variability, and the desired level of community control. Ecological stability denotes the capacity of a community to maintain structure and function over time, encompassing persistence, resistance to perturbation, and resilience following disturbance [76]. In engineered consortia, stability emerges from the balance of cooperative and competitive interactions, reinforced by niche partitioning, cross‐feeding, spatial organization, and controlled population ratios [77, 78, 79]. Unlike monocultures, multispecies systems require explicit management not only of strain identity but of relative abundance and spatial arrangement, as diffusion constraints and local microenvironments shape interaction outcomes. Notably, spatial organization itself can function as an active stabilization mechanism. Experimental studies have shown that microscale spatial structuring can prevent competitive exclusion and promote long‐term coexistence, while engineered compartmentalization strategies such as microcapsules or structured biofilms can improve community robustness and population control [80, 81, 82].

Recent advances in synthetic ecology have translated ecological theory into quantitative design strategies [83, 84, 85, 86]. Model‐guided reconstruction of mutualism, competition, commensalism, and non‐transitive motifs has enabled predictable coexistence architectures in simplified systems [77, 87]. Engineered cross‐feeding loops, auxotrophy‐based dependencies, and feedback‐regulated kill‐switches have been used to stabilize population ratios and prevent competitive exclusion [74, 88, 89]. For example, engineered auxotrophic cross‐feeding systems have been used to establish stable metabolic interdependencies and maintain controlled population ratios in both bacterial and yeast consortia, while synthetic four‐species communities based on amino‐acid exchange have demonstrated increased population evenness across diverse environmental conditions, including gut‐associated ecosystems [35, 74, 90]. However, engineered stability remains fragile. Cheaters can exploit cooperative networks, evolutionary drift can erode designed dependencies, and interaction outcomes frequently shift across environmental contexts [77, 83, 85]. Moreover, higher‐order interactions often reshape system‐level behavior beyond pairwise predictions [79, 83, 91, 92], underscoring the need for system‐scale evaluation rather than reductionist interaction mapping.

Natural fermentation ecosystems provide instructive parallels. Systems such as wine, beer, cocoa, kombucha, and kefir illustrate recurrent ecological motifs including niche complementarity, metabolic succession, spatial stratification, and reciprocal metabolite exchange [93, 94, 95, 96, 97, 98]. Yeasts, lactic acid bacteria, and acetic acid bacteria partition metabolic roles while physicochemical gradients structure coexistence. These naturally assembled consortia demonstrate that stable functionality arises from distributed roles embedded within environmental constraints rather than centralized control. These ecological principles have also been successfully translated into engineered systems, where division‐of‐labour architectures, nutrient partitioning strategies, and spatial segregation have been deliberately implemented to stabilize population dynamics, improve coexistence, and enhance functional robustness across synthetic communities [70, 99]. Importantly, such ecosystems also provide actionable ecological design principles for synthetic consortia engineering, including the importance of niche complementarity, metabolite handoffs, spatial organization, dynamic succession, and environmentally mediated stabilization mechanisms. These principles can guide engineering decisions regarding species selection, substrate partitioning, sequential pathway organization, spatial compartmentalization, and stabilization strategies aimed at reducing competitive exclusion and improving long‐term coexistence. Rather than relying exclusively on deterministic regulation, these systems illustrate how robust functionality can emerge from distributed ecological interactions operating under physicochemical constraints.

Translating these principles into synthetic systems requires explicit attention to metabolite transport and flux compatibility. Division of labor frequently fails not at the genetic level but at the interface of metabolite exchange. Transport bottlenecks such as vacuolar sequestration of S‐adenosylmethionine (SAM) [100, 101, 102], limited diffusion of 3‐dehydroshikimate requiring engineered permeases [103], or membrane permeability constraints during vitamin K_2_ biosynthesis in coculture [104] illustrate how physical and biochemical barriers can destabilize otherwise well‐designed interactions. Effective synthetic ecology therefore demands integration of metabolic engineering with transport optimization and ecological modeling.

Within the systems‐level framework adopted here, ecological structuring defines the stability layer of community engineering. Robust consortia emerge not from static interaction maps alone, but from distributed resilience, redundancy, spatial organization, and adaptive feedback. Future progress will depend on incorporating higher‐order interaction motifs into predictive models and integrating real‐time monitoring systems capable of detecting compositional drift and functional imbalance [83]. Such convergence between ecological theory, quantitative modeling, and adaptive control is essential for achieving scalable and durable synthetic microbial consortia.

## 6 Data Integration and Modeling: The Predictive Coordination Layer of Synthetic Consortia

**FIGURE 4:**
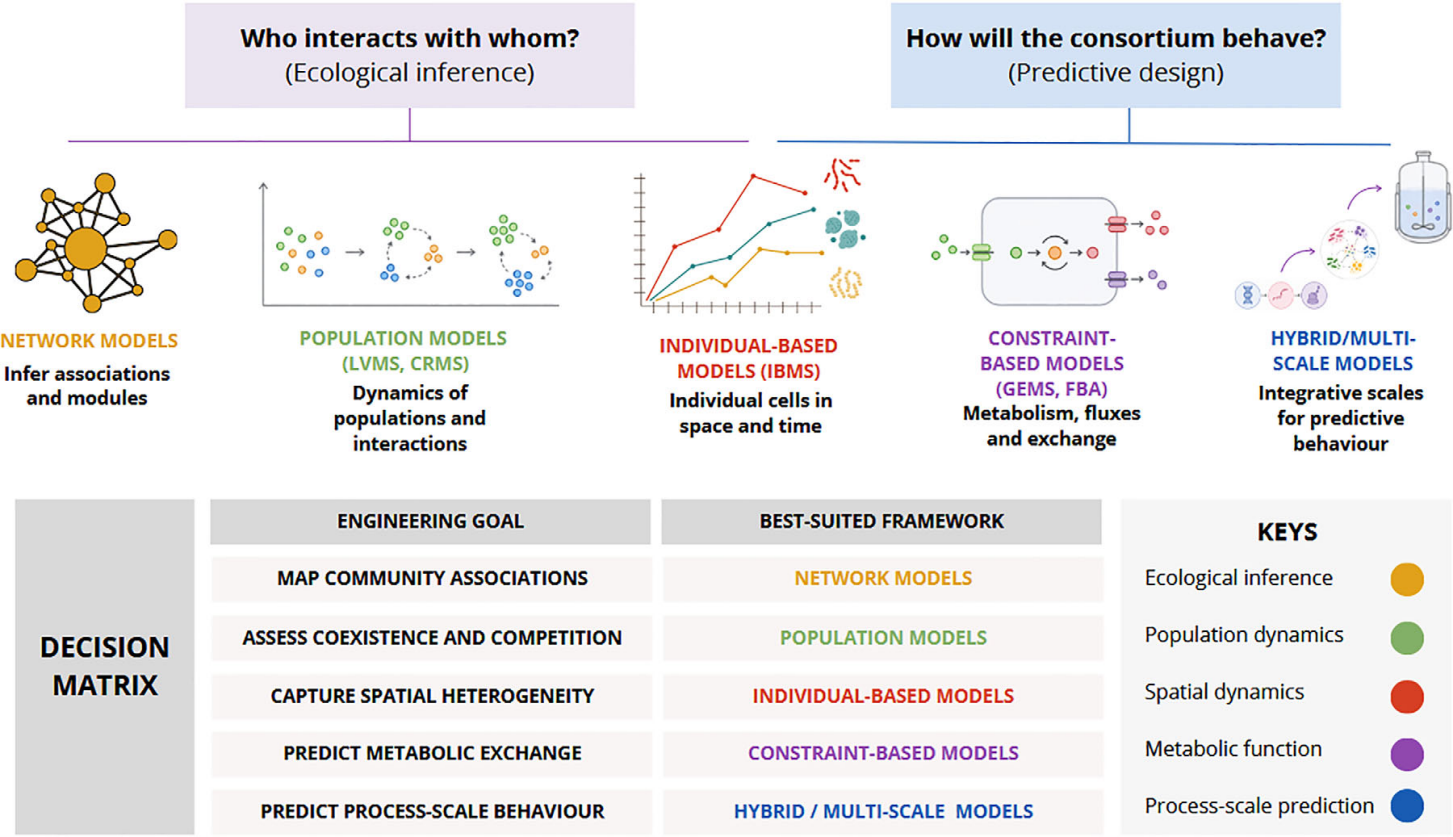
Modelling frameworks for application‐dependent consortium engineering. Conceptual overview of major modelling frameworks used to support the design, analysis, and optimization of synthetic microbial consortia. Different modelling approaches are suited to distinct engineering questions and operate at different levels of biological organization. Network‐based models support ecological inference by identifying community associations and candidate interaction structures. Population‐based models describe coexistence dynamics, competition, and community stability across interacting populations. Individual‐based models capture spatial organization, stochasticity, and cell‐level heterogeneity in structured environments. Constraint‐based models predict metabolic fluxes, cross‐feeding interactions, and division of labour, whereas hybrid and multi‐scale frameworks integrate multiple modelling resolutions to support process‐scale prediction and consortium design. The decision matrix illustrates how different engineering objectives may require different modelling strategies, highlighting that model selection should be guided by the specific biological question, desired predictive capability, and level of system complexity.

Modeling constitutes the predictive coordination layer of multimicrobial engineering, enabling the integration of metabolic, ecological, spatial, and temporal information into rational design strategies for synthetic microbial consortia. Unlike intracellular circuit engineering, where behaviour is often constrained within relatively defined cellular architectures, multispecies systems involve distributed interactions across populations operating under dynamic environmental conditions. Consequently, effective consortium engineering frequently requires computational frameworks capable of predicting coexistence, metabolic exchange, synchronization dynamics, spatial organization, and process‐scale behaviour [105, 106, 107, 108]. Different engineering objectives require distinct modeling resolutions, data requirements, computational complexity, and predictive capabilities. Consequently, modeling serves not only to describe community behaviour but also to guide engineering decisions regarding species selection, interaction design, environmental configuration, and process optimization. Figure 4 summarizes representative modeling frameworks frequently used in synthetic microbial consortia engineering together with their principal scales, predictive capabilities, and operational limitations.

Network‐based models, for example, provide low‐resolution but computationally accessible approaches for inferring ecological organization within microbial communities. By analysing statistically non‐random co‐occurrence patterns among taxa or functional traits, these frameworks can identify candidate interactions, keystone populations, or potentially compatible ecological modules [109]. Such approaches are particularly useful during early‐stage consortium design or exploratory screening when mechanistic information remains limited. However, network models are fundamentally correlative and often lack explicit mechanistic representation of metabolic exchange, environmental dependency, or causal interaction dynamics, limiting their predictive power under changing conditions.

Population‐based frameworks such as Lotka–Volterra models (LVMs) and consumer‐resource models (CRMs) are commonly used to describe coexistence dynamics, competitive interactions, and resource partitioning across populations [110, 111, 112]. LVMs capture growth, inhibition, and coexistence relationships through interaction coefficients, whereas CRMs explicitly incorporate shared substrate utilization and resource transformation dynamics [113, 114, 115]. These models are particularly useful for evaluating ecological stability, competitive exclusion, niche differentiation, and community resilience under fluctuating environmental conditions. Recent extensions integrating adaptive uptake strategies, flexible stoichiometry, and environmentally responsive interactions further improve their capacity to represent metabolic plasticity in dynamic ecosystems [60, 116]. Nevertheless, population‐based approaches frequently face parameter identifiability limitations, particularly when interaction coefficients vary across environmental contexts or when experimental observables incompletely capture intracellular physiological states [112].

Individual‐based models (IBMs) provide higher spatial and mechanistic resolution by simulating the behaviour of individual cells within heterogeneous environments [117, 118, 119, 120]. These frameworks can incorporate stochasticity, diffusion gradients, local metabolite exchange, biofilm formation, and spatially constrained interactions, making them especially valuable for studying structured microbial communities and emergent synchronization phenomena [118, 121]. IBMs are particularly relevant when spatial organization strongly influences consortium behaviour, such as in biofilms, encapsulated systems, gut‐associated communities, or surface‐colonizing populations. However, these models are computationally demanding and often require large parameters that may be difficult to obtain experimentally. Increasing model complexity can therefore reduce parameter identifiability and limit predictive robustness under process‐scale conditions.

Constraint‐based metabolic models built on genome‐scale metabolic models (GEMs) and flux balance analysis (FBA) provide high‐resolution descriptions of intracellular metabolism and metabolic exchange [105, 107, 122, 123]. Originally developed for single organisms, these frameworks are now increasingly extended to microbial communities, enabling quantitative prediction of cross‐feeding interactions, metabolic division of labour, substrate allocation, and pathway compatibility [124, 125, 126, 127]. Several studies have successfully used GEMs and FBA‐based approaches to design and optimize synthetic microbial consortia by predicting metabolite exchange networks, identifying compatible community members, and defining media compositions that promote mutualistic or commensal interactions. For example, computational ecosystem design frameworks have been used to predict environmental conditions that induce stable metabolic cooperation between species, thereby reducing experimental trial‐and‐error during consortium assembly [128, 129]. GEM‐based approaches are particularly useful when consortium engineering objectives depend on optimizing metabolic flux distribution or balancing biosynthetic burden across populations. Their integration with metagenomics, transcriptomics, proteomics, and metabolomics data can substantially improve flux prediction and pathway‐level interpretation [122, 130, 131, 132]. However, GEM‐based approaches typically rely on steady‐state assumptions, incomplete metabolic annotations, and simplified transport representations that may limit predictive accuracy during real‐world implementation [133].

A major frontier in multimicrobial engineering lies in the hybrid integration of modeling scales. Increasingly, GEM‐derived metabolic predictions are coupled with ecological, dynamic, or spatial frameworks including CRMs, Lotka–Volterra systems, network models, or IBMs [134, 135]. Such hybrid approaches aim to connect intracellular metabolism with population‐level ecological dynamics and environmental interactions, thereby improving prediction of coexistence, division of labour, and community‐level functionality [136, 137]. Experimental validation of hybrid modeling approaches has demonstrated their ability to predict community composition, metabolic interactions, and spatial organization across engineered microbial systems. For example, dynamic flux balance analysis coupled with spatial diffusion models successfully predicted species ratios and equilibrium states in engineered mutualistic consortia, while platforms such as COMETS have enabled simulation of metabolically interacting communities under spatially structured and evolving environmental conditions [137, 138]. These examples illustrate how modeling can move beyond descriptive analysis to actively guide consortium design and optimization.

The practical implementation of multi‐scale integration remains challenging. Parameter uncertainty can propagate across modeling layers, while models calibrated under laboratory conditions often exhibit limited transferability across cultivation scales and environmental contexts due to changes in diffusion constraints, mixing regimes, oxygen transfer, and spatial heterogeneity [139]. These challenges become particularly relevant in industrial bioprocesses, where oxygen gradients, substrate heterogeneity, delayed signal propagation, and uneven mixing can substantially alter community dynamics [140]. Consequently, hybrid modeling should not be interpreted as a universally predictive solution, but rather as an evolving engineering strategy whose performance depends on data quality, system complexity, and operational objectives. Increasing availability of multi‐omics and process‐monitoring data, however, offers new opportunities to improve model parameterization, calibration, and predictive accuracy across scales.

Real‐time monitoring and adaptive recalibration in particular are becoming increasingly valuable for obtaining predictive control in synthetic consortia. Technologies such as online flow cytometry [141], optical biosensors [142], Raman spectroscopy [143], and metabolite‐sensitive sensing platforms enable continuous characterization of growth dynamics, population ratios, and metabolic states during bioprocess operation. When integrated with computational frameworks, these data streams support iterative design‐build‐test‐learn cycles in which model predictions are continuously refined through experimental feedback. These developments are progressively transforming modeling from a purely predictive activity into an adaptive engineering layer capable of continuously refining consortium design and operational control through experimental feedback. Such adaptive strategies may improve robustness under fluctuating conditions while enabling dynamic process control and early detection of compositional drift or functional instability.

Different modeling approaches provide distinct balances between mechanistic resolution, computational tractability, experimental requirements, and predictive capability. Effective modeling strategies consequently depend on engineering objectives, environmental complexity, available experimental data, and the degree of predictive control required for a given application. By integrating metabolic, ecological, spatial, and process‐scale information, modeling frameworks provide an essential foundation for the rational design, stabilization, and optimization of synthetic microbial consortia across industrial, environmental, and therapeutic systems.

## 7 A Modular Systems‐Engineering Blueprint for Synthetic Microbial Consortia

The coordination layers discussed throughout this Perspective should not be interpreted as universally required components of synthetic microbial consortia, nor as elements that must be implemented in a predefined sequence. Rather, they represent modular engineering dimensions that can be selectively combined according to the functional objectives, environmental complexity, and operational constraints of a given application. The central challenge of consortium engineering is therefore not the implementation of all available coordination strategies, but the rational identification of which coordination layers are necessary to achieve a desired function while maintaining robustness, scalability, and controllability.

A central design variable is the engineering objective itself. Different applications impose distinct coordination requirements across microbial populations and therefore demand different combinations of communication, temporal regulation, ecological structuring, and predictive modeling. As summarized in Table 1, the relative importance of these coordination layers varies considerably depending on the functional demands of the system. For example, metabolite production systems are often driven by metabolic compatibility, division of labour, and flux optimization, whereas food fermentations rely more heavily on ecological succession and community stability. Environmental remediation typically prioritizes ecological persistence and resilience under fluctuating conditions, while living therapeutics require tighter control over microbial behaviour, host responsiveness, and ecological integration. At the opposite end of the spectrum, precision biomanufacturing may require simultaneous implementation of multiple coordination layers to enable adaptive control, real‐time optimization, and predictive process management [75]. These examples illustrate that consortium engineering is fundamentally application‐dependent and that no single coordination architecture is universally optimal.

**TABLE 1:** Application‐dependent decision matrix for selecting coordination layers in synthetic microbial consortia. Representative engineering objectives are mapped to their expected coordination requirements, key engineering constraints, and primary design rationales, illustrating how different applications demand distinct combinations of communication, temporal coordination, ecological structuring, and predictive modelling. The relative importance of each coordination layer is indicative rather than prescriptive and depends on the engineering objective, environmental complexity, and desired level of system control.

Engineering objective	Communication	Temporal coordination	Ecological structuring	Predictive modelling	Key engineering requirements	Primary design rationale
Metabolite production	Moderate–High	Low–Moderate	Moderate	High	Metabolic compatibility, division of labour, flux balancing, productivity optimization	Metabolite production is primarily driven by metabolic compatibility, division of labour, and flux optimization across consortium members. Communication through metabolite exchange often supports pathway coordination, whereas explicit temporal regulation and advanced ecological stabilization become more important only in complex or highly interdependent production systems.
Food fermentation	Low–Moderate	Low	Very High	Moderate–High	Functional succession, community stability, product consistency & reproducibility, process robustness	Community functionality is primarily governed by ecological succession, niche occupation, and interspecies metabolic interactions. Temporal organization often emerges naturally from succession dynamics, while explicit communication and temporal control are generally less important than maintaining stable community structure and reproducible ecological trajectories.
Environmental remediation	Low–Moderate	Low	Very High	High	Ecological persistence, distributed degradation, community integration, resilience to environmental fluctuations	Successful remediation depends on the persistence and ecological integration of functional populations within heterogeneous and fluctuating environments. Consequently, ecological robustness and predictive understanding of community interactions are often more important than explicit communication or temporal synchronization mechanisms.
Living therapeutics	High	Moderate	Very High	High	Ecological integration, host responsiveness, functional stability, safety, and population control	Host‐associated applications require tight control of microbial behaviour, environmental responsiveness, safety, and ecological integration within complex host ecosystems. Communication systems often support context‐dependent responses, while ecological stabilization and predictive modelling are critical for maintaining robust functionality.
Precision bio‐manufacturing	High	High	High	High	Sequential transformations, adaptive control, real‐time optimization, process robustness, and scale‐up performance	Precision biomanufacturing requires coordinated regulation across multiple organizational layers, including communication, temporal control, ecological stability, and predictive modelling. The integration of real‐time monitoring and adaptive process control enables dynamic optimization of community performance under changing operational conditions.

*Coordination requirements are indicative rather than prescriptive and depend on system complexity, environmental variability, and the desired degree of control.

The selection of coordination layers should therefore be driven by engineering requirements rather than by assumptions regarding consortium complexity. In many cases, robust functionality can emerge from relatively simple architectures based on metabolic complementarity, constitutive cross‐feeding, or ecological niche partitioning alone. Conversely, applications involving sequential transformations, adaptive responses, synchronized activity, or dynamic environmental interactions may require additional communication, temporal coordination, or predictive control layers [144]. The objective is not to maximize coordination complexity, but to deploy only those layers that contribute meaningfully to system performance.

Regardless of the selected coordination architecture, microbial consortia frequently operate under changing environmental conditions. Stability should therefore be evaluated as a dynamic property rather than as a fixed endpoint. Population drift, transport limitations, environmental heterogeneity, evolutionary adaptation, and process‐scale constraints may progressively alter consortium behaviour. Consequently, experimental validation and monitoring remain essential components of consortium engineering. Real‐time sensing technologies, flow cytometry, metabolomics, biosensors, and adaptive design‐build‐test‐learn cycles can support iterative refinement while enabling early detection of instability, compositional drift, or performance loss [145].

The framework proposed here provides a structured decision framework for selecting, integrating, and evaluating coordination layers according to application‐specific requirements. By reframing consortium engineering as the rational integration of modular coordination layers, this blueprint provides a practical systems‐engineering foundation for the design, stabilization, and optimization of synthetic microbial consortia across industrial, environmental, and therapeutic applications.

## 8 Conclusion

Synthetic biology is increasingly moving beyond the engineering of individual microorganisms toward the design of coordinated multicellular systems. As this transition accelerates, engineering frameworks based solely on intracellular genetic circuits become insufficient to capture the ecological, temporal, spatial, and metabolic interactions that shape community‐level behaviour.

In this Perspective, we propose a symphony‐inspired systems‐engineering framework that reframes synthetic microbial consortia as distributed biological systems whose functionality emerges from the coordinated interaction of multiple engineering dimensions. Rather than prescribing a universal architecture, the framework provides a modular approach for selecting and integrating coordination layers according to application‐specific requirements, environmental complexity, and desired levels of control. A major challenge moving forward will be improving the predictability, scalability, and long‐term stability of engineered microbial communities across laboratory, industrial, environmental, and therapeutic settings. Addressing these challenges will require closer integration of ecological theory, synthetic biology, predictive modeling, real‐time monitoring, and bioprocess engineering. Ultimately, advancing from isolated genetic control toward coordinated systems design may provide a foundation for building more robust, adaptive, and reliable microbial communities capable of addressing increasingly complex biotechnological challenges.

## Author Contributions

Conceptualization: MF‐N. Funding acquisition: MF‐N and SM‐G. Investigation: MF‐N, LAW, JMG, DB‐T, SM‐G, GDM, Z‐uI, RvT‐P, VRM, and RL‐A. Visualization: MF‐N, DB‐T, and SM‐G. Writing – original draft: MF‐N, LAW, JMG, DB‐T, SM‐G, GDM, Z‐uI, RvT‐P, VRM, and RL‐A. Writing – review & editing: MF‐N, LAW, JMG, DB‐T, SM‐G, GDM, Z‐uI, RvT‐P, VRM, and RL‐A. All authors have read and approved the final version of the manuscript.

## Funding

IATA‐CSIC received funding from the Spanish Ministry of Science and Innovation, ref. MCIN/AEI/10.13039/501100011033, as a ‘Severo Ochoa’ Centre of Excellence (CEX2021‐001189‐S). Vanessa Rossetto Marcelino was supported by the Spanish Ministry of Science and Innovation through the Ramón y Cajal program (Grant RYC2023‐042907‐I). Miguel Fernández‐Niño and Silvia Moriano‐Gutiérrez were supported by the internal IATA‐CSIC project IMPULSA‐IATA2025‐2 (Severo Ochoa).

## Conflicts of Interest

The authors declare no conflicts of interests.

## Data Availability

This article does not report new experimental or computational data. All information discussed in this Perspective is derived from previously published studies, which are cited within the article.
